# Proteus syndrome: evaluation of the immunological profile

**DOI:** 10.1186/s13023-015-0381-z

**Published:** 2016-01-13

**Authors:** Vassilios Lougaris, Vincenzo Salpietro, Maricia Cutrupi, Manuela Baronio, Daniele Moratto, M. R. Pizzino, Kshitij Mankad, Silvana Briuglia, Carmelo Salpietro, Alessandro Plebani

**Affiliations:** Pediatrics Clinic and Institute for Molecular Medicine A. Nocivelli, Department of Clinical and Experimental Sciences, University of Brescia, Piazzale Spedali Civili 1, Brescia, 25123 Italy; Department of Pediatrics, Unit of Pediatric Genetics and Immunology, University of Messina, Messina, Italy; Institute for Molecular Medicine A. Nocivelli, and Department of Pathology, Laboratory of Genetic Disorders of Childhood, Department of Molecular and Translational Medicine, University of Brescia, Spedali Civili di Brescia, Brescia, Italy; Department of Radiology, Great Ormond Street Hospital for Children National Health Service Foundation Trust, London, UK

**Keywords:** Proteus syndrome, Overgrowth, B cells, T cells

## Abstract

Proteus syndrome (PS) is an extremely rare and complex disease characterized by malformations and overgrowth of different tissues. Prognosis of affected patients may be complicated by premature death, mostly due to pulmonary embolism and respiratory failure. To date, immunological data in Proteus syndrome are scarse.

We report on the novel immunologic findings of a 15 years old girl affected with PS. Detailed T and B cell evaluation revealed maturational alterations for both subsets and functional hyperactivation for the latter. Such findings have not been reported previously in PS and may be the spy of more complex immune abnormalities in this syndrome.

## Correspondence

The Proteus syndrome (PS) is an extremely rare and complex disease characterized by malformations and overgrowth of various tissues, mainly connective tissue, bone, skin, and central nervous system, although any tissue may be involved [1–6]. Clinical manifestations are highly variable and the disproportionate overgrowth of tissue is usually asymmetrical and involves the arms, legs, hands, feet and digits. The complications of PS include hyperostosis, cerebriform connective tissue progressive, skeletal deformities, benign and malignant tumors, capillary vascular malformations and deep venous thrombosis with pulmonary embolism.

The syndrome has an incidence of less than 1 per 1,000.000 live births and is estimated that 120 individuals with PS are currently alive worldwide [3–5]. Newborns with Proteus syndrome have few or no signs of the condition, and overgrowth becomes apparent between 6 and 18 months of life, getting more severe with age [6]. Lesions appear to be distributed in a mosaic manner and have a progressive evolution. Recently, a mosaic activating mutation in AKT1 was reported to be associated with PS [7].

Published immunological data for PS are scarse: in fact, only one patient has been reported to date with mild hypogammaglobulinemia and lymphopenia leading to reduction of total T and B cell numbers [8].

## Case report

**Fig. 1:**
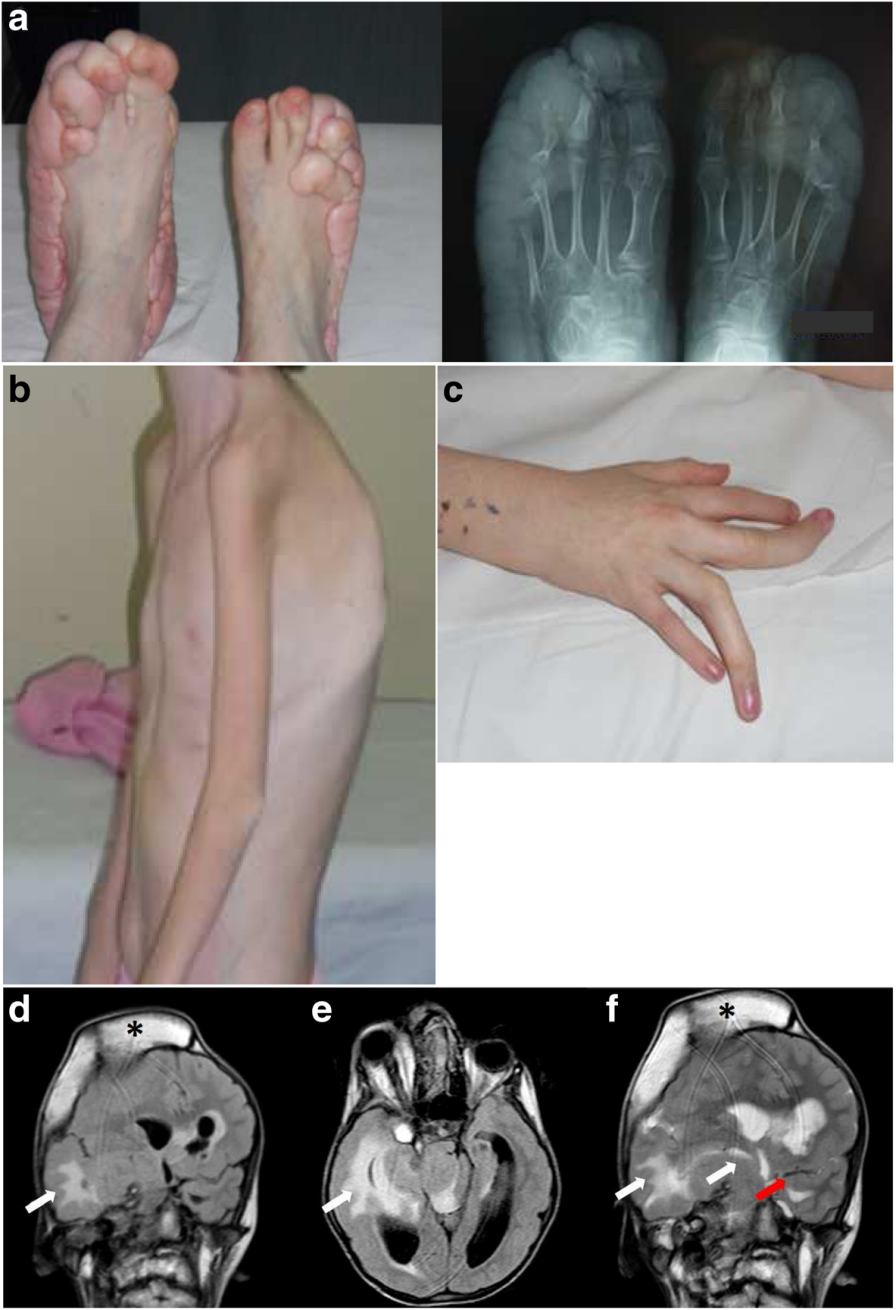
Clinical and radiologic presentation of the index PS patient. **a** Overgrowth of the feet (*left panel*) with radiologic confirmation of bone overgrowth, especially of the left foot (*righ panel*). **b** Severe cyphoscoliosis of the index patient. **c** Length discrepancy and macrodactyly in the III and IV fingers of right hand from the index patient. **d e** and **f** Coronal flair (**d**) and axial flair (**e**) weighted images demonstrated hyperostosis of the right fronto-parietal cranial vault (*black asterisk*) with proptosis of the right eye and extensive malformation of cortical development involving predominantly the right cerebral hemisphere but also part of the left cerebral hemisphere. There is an extensive white matter signal abnormality of both hemispheres (*white arrows*); there is also an associated malformation of the brainstem. Coronal T2 weighted images (**f**) show hyperostosis of the right fronto-parietal cranial vault (*black asterisk*), extensive malformation of the right hemisphere with white matter changes in the periventricular region (*white arrows*); malformation of cortex in the left perisylvian region (*red arrow*)

We report the case of a 15 years old girl with Proteus syndrome and describe novel immunological findings in PS. The index patient, born to non consanguineous parents, was born via caesarian section at 36 weeks of gestation, with normal weight and length (3500 g and 50 cm, respectively). Diagnosis of PS was made at the age of 2 years, with progressive overgrowth over time. The index patient presented multiple lesions affecting the central nervous and circulatory system, lung, skeleton, limbs and abdominal organs. The phenotypic examination showed dolichocephaly, hyperostosis of the skull in the right fronto-parietal area, facial dysmorphic features with facial asymmetry and ptosis of right eye, depressed nasal bridge, wide and anteverted nares, oligodontia and multiple caries, long neck. The patient presented thin skin, adipose dysregulation with poorly represented subcutaneous adipose and a disproportionate, asymmetric overgrowth of the limbs. The index patient presented important overgrowth of the feet (Fig. 1a, *left panel*) with greater growth of the calcaneus and cuboid, especially of the left foot (Fig. 1a, *right panel*). The patient also presented severe kyphoscoliosis (Fig. 1b) and length discrepancy and macrodactyly in the III and IV fingers of right hand (Fig. 1c), emphysema, pulmonary fibrosis, syringomyelic cavity of about 2 cm in the section C3-D4, and ovarian cystadenomas. During follow-up, the patient developed portal thrombosis at 12 years of age with portal splenic and mesenteric hypertension and liver atrophy and suffered from a transient ischemic attack (TIA) at the age of 13. Brain magnetic resonance imaging (MRI) showed cortical dysplasia with marked thickening of the skull in the right fronto-temporal (Fig. 1d, e and f) which increased in volume over 4 years reaching 44 mm with significant compression on the midbrain, brain parenchyma and cerebral edema, hydrocephalus and proptosis of the right eye (Fig. 1e). The significant progression of the lesions determined respiratory failure, coma, and subsequent death of the patient.

**Fig. 2:**
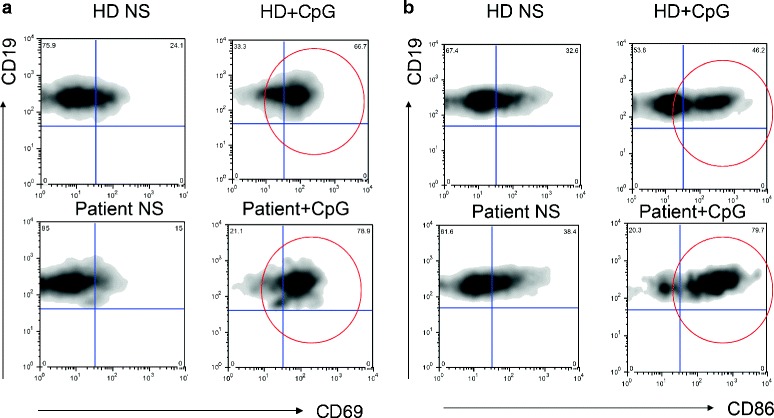
B cell *in vitro* activation from the index patient. **a** B cells from the index patient and a healthy control (HD) were stimulated with CpG and CD69 up-regulation was evaluated by flow-cytometry after overnight incubation. **b** B cells from the index patient and a healthy control (HD) were stimulated with CpG and CD86 up-regulation was evaluated by flow-cytometry after overnight incubation. Red circles depict the activated cell populations

Immunological evaluation of the index patients revealed several novel findings. Immunoglobulin serum levels were within normal range, in contrast with previously reported data [8] in the presence of valid humoral response to vaccinations (Table 1). Interestingly, both the index patient and the single patient reported previously [8] were lymphopenic (Table 1), with consequently reduced peripheral total T and B cell numbers (Table 1), without however a significant history of infections. Thus, a more detailed evaluation of the peripheral lymphocyte subsets from the index PS patient was performed and revealed, within the T cell population, normal thymic output as defined by normal naive (CD45RA^+^CCR7^+^) and recent thymic emigrants (RTE) (CD45RA^+^CCR7^+^CD31^+^) CD4 T cell percentages, an increase in the Terminally differentiated CD45RA^+^CCR7^−^ CD4 T cells and a reduction of the CD8 T cells (Table 1). The B cell population showed a reduction in the RBE (Recent Bone Marrow Emigrants: CD38^hi^CD21^dim/lo^CD27^−^) subset and in the IgM memory subset (IgD^+^CD27^+)^, while all other subsets appeared normally distributed in terms of percentages (Table 1). No alterations were observed in the natural killer (NK) cell subsets. T and B cell activation was evaluated by in vitro stimulation with phytohemaggluttinin (PHA) and CpG respectively and measured by the up-regulation of surface activation markers. T cell activation upon (PHA) stimulation as measured by CD69 up-regulation was similar to that of the healthy control (data not shown). Patient’s B cells on the other hand showed an increased up-regulation of CD69 and CD86 upon CpG stimulation when compared to the healthy control (Fig. 2a and b respectively), suggestive of B cell hyper responsiveness in PS.

**Table 1:** Immunological evaluation of the index PS patient

Patient code	Index patient	Normal range for age (index patient)	Published patient [8]
Sex	Female		Male
Age (onset)	2 years		n.a.
Age (evaluation)	14 years		10 years
IgG (mg/dl)	1110	(604–1909)	280
IgA (mg/dl)	100	(61–301)	40
IgM (mg/dl)	105	(59–297)	50
Anti-tetanus toxoid antibodies (UI/ml)	0,15		
Anti-diphtheria antibodies (UI/ml)	0,12		
Total lymphocyte count (/mm^3^)	736 ↓↓	(1340–3173)	470
T cell counts/ml	437 ↓↓	(954–2332)	100
B cell counts/ml	164 ↓	(173–685)	100
	% (absolute cell number/μl)	Normal percentage range for age (range of absolute cell number/μl for age) (index patient)	
T cells (CD3^+^)	59,3 ↓ (437 ↓↓)	60,5–79,8 (954–2332)	n.a.
CD3^+^CD4^+^	43,2 (318 ↓↓)	30,3–48,3 (635–1334)	n.a.
Naive (CD45RA^+^CCR7^+^)	49,9 (159 ↓)	34,3–74,6 (276–828)	n.a.
RTE (CD45RA^+^CCR7^+^CD31^+^)	39,9 (127)	21,1–63,5 (21–699)	n.a.
Central memory (CD45RA^−^CCR7^+^)	22,6 (72 ↓)	13,0–43,5 (107–420)	n.a.
Effector memory (CD45RA^−^CCR7^−^)	19,5 (62 ↓)	8,5–28,1 (95–261)	n.a.
Terminally differentiated (CD45RA^+^CCR7^−^)	7,6 ↑ (24)	0,7–6,6 (6–61)	n.a.
CD3^+^CD8^+^	9,1 ↓↓ (40 ↓↓↓)	13,8–37,5 (276–1035)	n.a.
Naive (CD45RA^+^CCR7^+^)	58,5 (23,4 ↓↓↓)	26,7–72,9 (117–454)	n.a.
Central memory (CD45RA^−^CCR7^+^)	10,2 (4 ↓)	1,2–11,6 (8–85)	n.a.
Effector memory (CD45RA^−^CCR7^−^)	15,6 (6 ↓↓)	6,0–53,6 (19–536)	n.a.
Terminally differentiated (CD45RA^+^CCR7^−^)	15,5 (6 ↓↓)	3,9–72,0 (13–699)	n.a.
TCR γ/δ	3,3 (1)	0,5–21,5	n.a.
B cells (CD19^+^)	22,3 (164 ↓)	5,7–19,7 (173–685)	n.a.
RBE (CD38^hi^CD21^dim/lo^CD27^−^)	12,1 ↓ (20)	15,0–35,3 (20–179)	n.a.
Naive (CD38^dim/lo^CD21^hi^CD27^−^)	66,1 (108)	33,8–79,6 (40–398)	n.a.
CD19^hi^CD21^lo^	6,3 (10)	1,1–10 (5–17)	n.a.
Switched memory (IgD^−^CD27^+^)	8,8 (14)	2,7–20,6 (7–45)	n.a.
IgM memory (IgD^+^CD27^+^)	2,5 ↓ (4 ↓↓)	3,5–24,1 (10–49)	n.a.
Terminally differentiated (CD38^hi^CD27^hi^CD21^lo^)	0,55 (0,5)	0,16–8,70 (0,4–11)	n.a.
Plasmacells (CD38^hi^CD20^−^CD138^+^)	0,21 (0,5)	0,04–3,20 (0,1–5,2)	n.a.
NK cells (CD3^−^CD16^+^CD56^+^)	11,0 (81 ↓)	4,6–27,8 (111–539)	n.a.

(Comparison with the only published immunological data from a single PS patient is included where data were available)

Our immunological data suggest that PS patients may present T and B cell alterations in terms of maturation and activation. This is a novel finding in PS and, together with the presence of lymphopenia, adds further complexity to this disorder. The in vitro hyper-responsiveness observed in B cells from the PS patient may be related to the role of AKT kinases in B cell biology in terms of apoptosis, survival and maturation, as observed in the animal model [9]. Unfortunately, and due to the premature death of the index patient, genetic analysis for the AKT1 mutation was not performed. Further studies in more PS patients would be of interest for better understanding the immune alterations in this rare and complex disorder.

## Ethics approval and consent to participate

The research presented here was performed in compliance with the Helsinki Declaration and was approved by the local Hospital Ethical Committee (Spedali Civili of Brescia (Italy) Ethical Committee). Written informed consent was obtained from the patient's parent for the publication of this report and any accompanying images.

## Abbreviations

MRI: magnetic resonance imaging
NK: natural killer
PHA: phytohemaggluttinin
PS: proteus syndrome
RBE: recent bone marrow emigrants
RTE: recent thymic emigrants
TIA: transient ischemic attack
